# Fast and Automatic Ultrasound Simulation from CT Images

**DOI:** 10.1155/2013/327613

**Published:** 2013-11-18

**Authors:** Weijian Cong, Jian Yang, Yue Liu, Yongtian Wang

**Affiliations:** Key Laboratory of Photoelectronic Imaging Technology and System of Ministry of Education of China, School of Optics and Electronics, Beijing Institute of Technology, Beijing 10081, China

## Abstract

Ultrasound is currently widely used in clinical diagnosis because of its fast and safe imaging principles. As the anatomical structures present in an ultrasound image are not as clear as CT or MRI. Physicians usually need advance clinical knowledge and experience to distinguish diseased tissues. Fast simulation of ultrasound provides a cost-effective way for the training and correlation of ultrasound and the anatomic structures. In this paper, a novel method is proposed for fast simulation of ultrasound from a CT image. A multiscale method is developed to enhance tubular structures so as to simulate the blood flow. The acoustic response of common tissues is generated by weighted integration of adjacent regions on the ultrasound propagation path in the CT image, from which parameters, including attenuation, reflection, scattering, and noise, are estimated simultaneously. The thin-plate spline interpolation method is employed to transform the simulation image between polar and rectangular coordinate systems. The Kaiser window function is utilized to produce integration and radial blurring effects of multiple transducer elements. Experimental results show that the developed method is very fast and effective, allowing realistic ultrasound to be fast generated. Given that the developed method is fully automatic, it can be utilized for ultrasound guided navigation in clinical practice and for training purpose.

## 1. Introduction

The imaging principle behind an ultrasound is that the ultrasound wave generates a different amount of reflection or refraction when accounting for different tissues inside the human body. Given that the shape, density, and structure of different organs vary, the amounts of wavelets that are reflected or refracted can be used to reconstruct the anatomical structure of human tissues. Based on the wave pattern and image features, combined with personal anatomical and pathological knowledge, the texture and pathological characteristics of a specific organ can be quantified for medical professionals.

Over the past decades, the ultrasound imaging technique has played increasingly important role in clinical diagnosis. As a fast and safe method of imaging, ultrasound is the most ideal imaging modality for real-time image-guided navigation in minimally intrusive surgery [1–3]. However, the ultrasound image is usually mixed with a high level of noise and the anatomical structure is not as clear as that in CT and MRI [4]. Hence, a successful ultrasound doctor has to possess a huge amount of anatomical knowledge, as well as considerable clinical experience. Currently, ultrasound clinical training is usually done under the guidance of experts who operate on real patients. Such training is time consuming and costly. Moreover, for some operations requiring careful manipulations, such as abscess drainage and tissue biopsy, incorrectly performed operations can cause great suffering to the patient or even lead to a number of complications. Comparably, the ultrasound simulation technique provides an economic and efficient way of observing and acquiring ultrasound images [5–7].

Currently, two categories of ultrasound simulation methods exist. The first involves the 3D ultrasound volume generated by a series of 2D ultrasound images, wherein the section slices of ultrasound images are generated from the location and direction of the ultrasound detector. Henry et al. [8] constructed the ultrasound volume from real images of a typical patient in offline preprocessing. The ultrasound image is then generated by considering both the position of the virtual probe and the pressure applied by this probe on the body. The system was successfully used to train physicians to detect deep venous thrombosis of the lower limbs. Weidenbach et al. [9] calculated a 2D echocardiographic image from preobtained 3D echocardiographic datasets that are registered with the heart model to achieve spatial and temporal congruency. The displayed 2D echocardiographic image is defined and controlled by the orientation of the virtual scan plane. Such a simulation method requires the 3D ultrasound volume data to be acquired in advance, thus guaranteeing good image quality and high-speed scanning of the image slice. However, this method cannot simulate the image outside the 3D volume data and 3D ultrasound images are also quite difficult to obtain using general ultrasound devices.

The second method involves the ultrasound being simulated from volume data, such as CT or MRI images. Shams et al. [10] simulated ultrasound images from 3D CT scans by breaking down computations into a preprocessing and a run-time phase. The preprocessing phase generates fixed-view 3D scattering images, whereas the run-time phase calculates view-dependent ultrasonic artifacts for a given aperture geometry and position within a volume of interest. Based on the method of Shams, Kutter et al. [11] used a ray-based model combined with speckle patterns derived from a preprocessed CT image to generate view-dependent ultrasonic effects, such as occlusions, large-scale reflections, and attenuation. In his method, Graphics Processing Unit (GPU) was introduced for speed acceleration. Reichl et al. [12] estimated ultrasound reflection properties of tissues and modified them into a more computationally efficient form. In addition, they provided a physically plausible simulation of ultrasound reflection, shadowing artifacts, speckle noise, and radial blurring. Compared with the ultrasound volume-based method, the source image is easy to obtain and the calculation is comparably robust for the CT- and MRI-based method [13]. However, given that the imaging principles are totally different for CT, MRI, and ultrasound, such kind of simulation is more complicated than the ultrasound volume-based method. Moreover, the method is time consuming during preprocessing and intensity calculations. On the other hand, the CT- and MRI-based method can conveniently obtain the ultrasound image at any angle and position and the simulated ultrasound can also be fused with the CT or MRI. Hence, the CT- and MRI-based method can provide a more comprehensive understanding of diseases.

In this paper, a novel method is developed for the simulation of an ultrasound image from CT volume datasets. A multiscale method is proposed to simulate blood flow and to enhance tubular structures in the CT image [14]. The thin-plate spline [15–17] interpolation method is utilized to transform images between the sector and rectangle diagram. Differences of adjacent regions in terms of radiation are subjected to weighted integration in the CT image to obtain a realistic simulation of the acoustic response of common tissues. Finally, based on reflection and attenuation principles of ultrasound, the Kaiser window function [18] is used to overlay simulated images from different transducer elements and the rectangular diagram is mapped into the sector diagram to guarantee a simulated ultrasound image with high validity and calculation speed.

The advantages of our algorithm are towfold: first, as the tubular structures in the CT image are strengthened by the multiscale enhancement method, the simulated vessel in the ultrasound is more realistic than the commonly used method. Second, as the response coefficient of ultrasound is calculated by the intensity differences of adjacent regions in the ultrasound propagation path, the complexity of the simulation procedure is greatly reduced.

## 2. Method

The developed method comprises the following four main parts.

**Figure 1:**
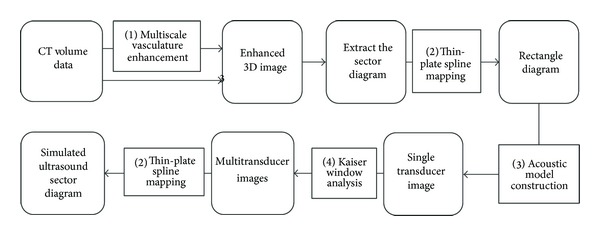
Simulation procedures and calculation modules.

1. *Multiscale Vascular Enhancement*. In this part, a multiscale method is employed to enhance tubular structures in the CT volume data. Through this process, the intensities unlikely to belong to vascular trees are effectively removed. The output image then used for following processing is a weighted integration of the source and the enhanced images.
2. *Thin-Plate Spline Mapping*. As ultrasound is generally presented as a sector diagram with a coordinate system that is different from the rectangular coordinate used for CT images, the thin-plate spline interpolation method is used for the transformation between sector and rectangular diagrams to achieve smooth mapping of both the diagrams.
3. *Acoustic Model Construction*. In this part, the acoustic model is constructed via the weighted function of adjacent regions on the ultrasound propagation path.
4. *Kaiser Window Analysis*. The ultrasound emitter is generally composed of multiple transducer elements. The Kaiser window filter is utilized to obtain a realistic simulation effect and to simulate fusion effects of all independent elements. In order to guarantee the clarity of the simulated ultrasound, a linear scaling method is applied to the final results to stretch the ultrasound intensity to a scale level of 256. The processing flow diagram is shown in Figure 1.

*(1)  Multiscale Vascular Enhancement*. When there is relative motion between the ultrasound source and the receiving body, the received signal frequency will be changed from the actual frequency transmitted from the source. Therefore, the vessels can be clearly imaged in ultrasound. For any CT image, the difference of CT values for vasculature and its neighboring tissues is almost negligible as no material is perfused in the focused part of the vasculature to enhance its visibility and subside the neighboring vessels as background during the whole procedure. Thus, it causes great difficulty in distinguishing the vasculature to be focused on and the neighboring tissues to be removed. Therefore, direct simulation of an ultrasound sector from a CT image cannot achieve realistic blood vessel visualization. In this paper, we utilize the multiscale enhancement method developed in [19] to strengthen vasculatures and then, by calculating intensify difference between adjacent voxels in the ultrasound propagation path, the response coefficient can be quantified.

The multiscale enhancement approach basically filters the tube like geometrical structures. Since there is a large variation in size of the vessels, so we need to define a measurement scale with a certain range. Basically to examine the local behavior of an image, *L*, its Taylor expansion in the neighborhood of a point *x*_0_ can be shown as

(1) $$\begin{array}{c} L\left(x_{0}+\delta x_{0},s\right)\approx L\left(x_{0},s\right)+\delta x_{0}^{T}\nabla_{0,s}+\delta x_{0}^{T}H_{0,s}\delta x_{0}, \end{array}$$

where ∇_0,*s*_ and *H*_0,*s*_ are the gradient vector and Hessian matrix of the image computed in *x*_0_ at scale *s*. To calculate these differential operators of *L*, we use the concepts of linear scale space theory. Here the differentiation is defined as a convolution with derivatives of Gaussians as

(2) $$\begin{array}{c} \frac{\partial }{\partial x}L\left(x,s\right)=s^{\gamma }L{\left(x\right)}^{*}\frac{\partial }{\partial x}G\left(x,s\right), \end{array}$$

where the *D*-dimensional Gaussian is defined as

(3) $$\begin{array}{c} G\left(x,s\right)=\frac{1}{{\sqrt{2\pi s^{2}}}^{D}}e^{-||x{||}^{2}/2a^{2}}. \end{array}$$

The parameter *γ* defines a family of normalized derivatives and helps in unbiased comparison of response of differential operators at various scales.

The idea behind eigenvalue evaluation of the Hessian is to extract the principal directions in which the local second order structure of the image can be decomposed. Three orthonormal directions are extracted by eigenvalue decomposition that is invariant up to a scaling factor when mapped by the Hessian matrix. Let *λ*_*k*_ be the eigenvalue with the *k*th smallest magnitude. In particular, a pixel belonging to a vessel region will be denoted by *λ*_1_ being small (ideally zero), *λ*_2_ and *λ*_3_ for a large magnitude and equal sign (the sign states the brightness or darkness of the pixel). To conclude, for an ideal tubular structure in a 3D image as

(4) $$\begin{array}{c} \left|\lambda_{1}\right|\approx 0\left|\lambda_{1}\right|\ll \left|\lambda_{2}\right|\;\lambda_{2}\approx \lambda_{3}. \end{array}$$

The polarity is indicated by signs of *λ*_2_ and *λ*_3_. In regions with high contrast compared to the background, the norm will become larger since at least one of the eigenvalues will be large. The following combination of the components can define a vesselness function:

(5) ν0(s)={0,λ2>0  or  λ3>0(1−exp⁡(−ℜA22α2)) ×exp⁡(−ℜB22β2) ×(1−exp⁡(−S22c2)),otherwise,

where *α*, *β*, and *c* are thresholds which control the sensitivity of the line filter to the measures *ℜ*_*A*_, *ℜ*_*B*_, and *s*. The vesselness measure is analyzed at different scales, *s*. For 2D images, we propose the following vesselness measure which follows from the same reasoning as in 3D:

(6) ν0(s)={0,λ2>0exp⁡(−ℜB22β2)(1−exp⁡(−S22c2)),otherwise,

where *ℜ*_*B*_ = *λ*_1_/*λ*_2_ is the boldness measure in 2D and accounts for the eccentricity of the second-order ellipse.

*(2)  Thin-Plate Spline Mapping*. As ultrasound and CT images are present as polar and rectangular coordinate systems, respectively, transformation between these two diagrams is necessary for the simulation processing. In this paper, the thin-plate spline interpolation method is utilized to achieve these transformations.

The basic idea of the thin-plate spline is that a space transformation can be decomposed into a global affine transformation and a local nonaffine warping component [20]. Assuming that we have two sets of corresponding points *p*_*i*_ and *q*_*i*_, *i* = 0,1,…, *n*, then, the energy function of the thin-plate spline can be defined as

(7) ETPS(f)=∑i=1n||pi−f(qi)||2+λ∬[(∂2f∂x2)2+2(∂2f∂x∂y)2       +(∂2f∂y2)2]dx dy,

where *f* is mapping function between point sets *p*_*i*_ and *q*_*i*_. The first term in the previous equation is the approaching probability between these two point sets, whereas the second term is a smoothness constraint, while *λ* indicates a different degree of warping. When *λ* is close to zero, corresponding points are matched exactly. For this energy function, a minimizing term *f*(*q*), *q* ∈ *R* ^2^ exists for any fixed *λ*, which can be formulated as:

(8) $$\begin{array}{c} f\left(q\right)=q\cdot A+\varphi \left(q\right)\cdot W, \end{array}$$

where *A* is a 3 × 3 affine transformation matrix and *φ*(*q*) is a 1 × *n* vector decided by the spline kernel, while *W* is a *n* × 3 non-affine warping matrix. When we combine (7) and (8), we have

(9) ETPS(A,W)=||U−V·A−ΨW||2+λ trace(WTΨW)dx dy,

where *U* and *V* are concatenated point sets of *p* and *q* and Ψ is a *n* × *n* matrix formed from the *φ*(*q*). Thus, *QR* decomposition can be utilized to separate the affine and non-affine warping space as follows:

(10) $$\begin{array}{c} M=\left[Q_{1}Q_{2}\right]\left(\begin{array}{c} R_{1}\\ 0 \end{array}\right)=Q_{1}R_{1}, \end{array}$$

where *M* is a *m* × *n* matrix with *m* ≥ *n*, *Q*_1_ is a *m* × *n* matrix, *Q*_2_ is *m* × (*m* − *n*) matrix, and *Q*_1_ and *Q*_2_ both have orthogonal columns, whereas *R*_1_ is an *n* × *n* upper triangular matrix. The final solution for *A* and *W* can be obtained as

(11) W=Q2(Q2TΨQ2+λI(N−3))−1Q2TU,A=R−1(Q2TV−ΨW).

Through thin-plate spline interpolation, the transformation between the polar and rectangular coordinate systems can be achieved. Although the thin-plate spline method is, to an extent, time consuming compared to the commonly used bilinear or trilinear interpolation methods, however, it guarantees comparative homogeneity in both radial and tangential directions. One common problem for the nonparametric mapping between polar and rectangular coordinate systems is that the resolution in tangential direction is homogeneous while it is reducing gradually radial direction from the center to the out part of the sector. The main merit of the proposed thin-plate spine mapping method is that it can keep maximum uniformity of the whole diagram.

**Figure 2:**
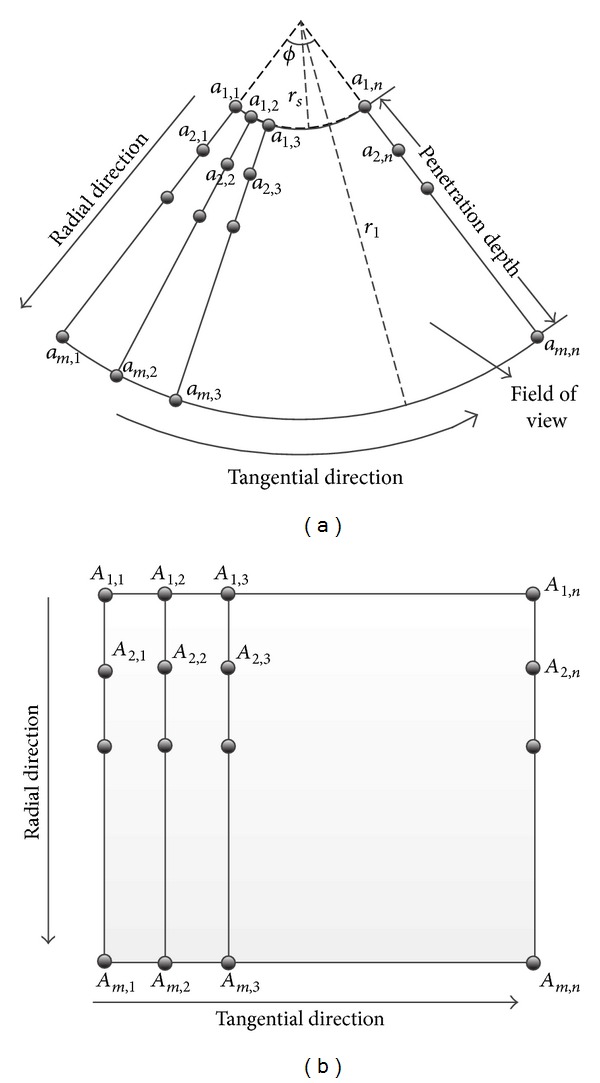
Relationship between sector and rectangle mapping.

Figure 2 shows the mapping principle between sector and rectangle. The ultrasound image is generally presented as sector, as shown in Figure 2(a), and the intersection angle *ϕ* is defined as Field of View (FOV) which is usually set as constant once the device is calibrated. The penetration depth of the ultrasound can be defined as the indepth distance between inner and outer circles with radius of *r*_*l*_ and *r*_*s*_, which is determined by the strength of acoustic wave.  Figure 2(b) shows the rectangle image section extracted from the CT image. In this figure, {*a*_1,2_, *a*_2,2_,…, *a*_*m*,2_} and {*A*_1,2_, *A*_2,2_,…, *A*_*m*,2_} are the constructed correspondences and *m* and *n* represent the number of sampling along radial and tangential directions. It is obvious that resolution of the simulated ultrasound is determined by *m* × *n*.

*(3)  Acoustic Model for Construction*. Large differences are observed in the acoustic resistances between different tissues. Thus, an ultrasound at interfaces of different tissues usually results in the occurrence of reflection, refraction, and absorption. If the resistance difference between two tissues is greater than 0.1%, the reflection will be produced [21]. The acoustic resistance *Z* of a certain organ can be calculated as *Z* = *ρc*, where *ρ* is density and *c* represents propagation speed of the ultrasound.

The reflection coefficient *α*_ref_ and transmission coefficient *α*_tran⁡_ on the interface of two organs with acoustic resistance of *Z*_1_ and *Z*_2_ can be calculated by the following equations [22]:

(12) αref(Z1,Z2)=UrefU=(Z2−Z1Z2+Z1)2,αtran⁡(Z1,Z2)=Utran⁡U=4Z1Z2(Z1+Z2)2=1−αref(Z1,Z2),

where *U*, *U*_ref_, and *U*_tran⁡_ are wave intensities of input ultrasound, reflected ultrasound, and transmitted ultrasound, respectively.

The reflection is generally produced on the interfaces of two organs. Hence, edge detection is imperative for acquiring boundary information. Currently, several stable edge detection methods exist, such as Roberts, Sobel, Prewitt, and LOG operators, which have been widely used in medical image processing. For these methods, the detection of the edge is based on the analysis of the intensity relationship of neighboring points. Moreover, if a certain angle exists between the propagation and edge directions, reflection will occur. If the propagation direction is parallel to the edge direction, the ultrasound will transmit directly, and no reflection occurs. Hence, the propagation angle must be considered in the calculation of the acoustic response.

However, a considerable amount of random speckles occur in the ultrasound image, and the correct noise generation is important for the realistic simulation of ultrasound images. When the scatter phenomenon of ultrasound is produced inside the human body, the backwaves with different phases generally interfere with one another. Hence, speckles are generated [23, 24]. Random noises are generated and superimposed onto the simulated image. As for the CT image, several factors, including amount of radiation, performance of data acquisition unit, and image reconstruction procedure, can also introduce noise in the simulated ultrasound [24].

Intensity differences of adjacent regions are used for the calculation of the response coefficient to obtain a realistic simulation of ultrasound. Specifically, the response coefficient of a certain region is determined by consecutive regions on the ultrasound propagation direction. The following three conditions have to be considered.

1. Adjacent regions are not on the interface. For such a condition, the calculation sample point is inside the same organ. Hence, the difference in the CT values of these two regions is small, yielding a small response coefficient.
2. Adjacent regions are on the edge of the interface. If the propagation direction is parallel to the edge direction, the adjacent regions will both be located on the edge, thus yielding small CT value variations. If the angle between the propagation and edge directions increases gradually, the CT value variation will increase and consequently increase the response coefficient. By this method, the interface effect of the response coefficient can be calculated only by adjacent regions, and the imaging angle between edge and ultrasound propagation directions need not be calculated.
3. Adjacent regions are on the noise area. In such situations, the difference in the CT values is usually large, thus yielding a large response coefficient. Therefore, the noises of the ultrasound can be simulated by the intensity difference in the CT image.

Acoustic resistance is generally known to be proportional to the CT value [25]. Hence, the weight *α*_ref_ of adjacent regions *I*(*x*_1_) and *I*(*x*_2_) can be written as

(13) $$\begin{array}{c} \alpha_{\text{ref}}={\left(\frac{I\left(x_{2}\right)-I\left(x_{1}\right)}{I\left(x_{2}\right)+I{\left(x_{1}\right)}_{1}}\right)}^{2}. \end{array}$$

However, bone-tissue interfaces reflect 43% and air-tissue interfaces reflect 99% of the incident beam [26]. Hence, (13) cannot be applied to tissues like bone and air.

*(4)  Kaiser Window Analysis*. For the acoustic response model, the strength of sound wave increases with the decrease of the angle *θ* between incident sound wave and surface normal at the interface, as can be shown by the Lambert cosine law [27] as follows:

(14) $$\begin{array}{c} U_{\text{out}}\left(\theta \right)=\alpha_{\text{ref}}\times U_{\text{in}}\times \cos\left(\theta \right), \end{array}$$

where *U*_in_ and *U*_out_ represent acoustic intensities before and after refraction at the medium interface. *α*_ref_ represents the refection coefficient and *θ* represents the intersection angle between input ultrasound and normal vector of the interface. When ultrasound is transmitted in the media, its energy decreases with the propagation distance. Such phenomenon is called ultrasound attenuation. As for ultrasound wave with given frequency, its energy attenuation follows the power law principle, which can be formulated as [28]

(15) $$\begin{array}{c} U\left(\alpha_{\text{ref}},d\right)=U_{\text{in}}\times e^{-2\alpha_{\text{ref}}d}, \end{array}$$

where *d* is the propagation distance, while *U*(*α*_ref_, *d*) represents the acoustic intensity after it has been propagated in the medium for a distance of *d*. According to the Lambert cosine law, the intensity of the acoustic response can be calculated as

(16) $$\begin{array}{c} U_{\text{out}}\left(\theta \right)=\alpha_{\text{ref}}\times U_{\text{in}}\times \cos\left(\theta \right)=\alpha_{\text{ref}}\times U_{\text{in}}\times \left|\overset{⃑}{r}\left(x\right)\cdot \overset{⃑}{n}\left(x\right)\right|, \end{array}$$

where $\overset{⃑}{r}(x)$ is the unit vector in the direction of the ultrasound beam, $\overset{⃑}{n}(x)$ is the surface normal at the interface, |·| is the absolute value operator. Then, the attenuation of the ultrasound can be obtained by the following equation:

(17) $$\begin{array}{c} U_{\text{out}}\left(\alpha_{\text{ref}},d,\theta \right)=\alpha_{\text{ref}}\times U_{\text{in}}\times \left|\overset{⃑}{r}\left(x\right)\cdot \overset{⃑}{n}\left(x\right)\right|\times e^{-2\alpha_{\text{ref}}d}. \end{array}$$

Suppose that multiple independent transducer elements are observed and the strength of each ultrasound is *U*_0_. The summary of the received ultrasound strength can be calculated as follows:

(18) Utotal=∑i=1nUi=∑i=1nαref×Uin×|r(x)·n(x)|×e−2αrefd=∑i=1nαref×Uin×cos⁡(θi)×e−2αref(d0/cos⁡⁡(θi)),

where *d*_0_ is the minimum distance among all the transducer element and sampling region *x*_*i*_ and *d* is the distance interval of adjacent transducer elements. Meanwhile, *θ*_*i*_ is the angle between transducer element and the sampling region *x*_*i*_, which can be written as

(19) $$\begin{array}{c} \theta_{i}=\arctan\left(\frac{n\times d}{d_{0}}\right), \end{array}$$

where *n* is the number of active elements of transducer and *ω*_*i*_ can be parameterized as *ω*_*i*_ = cos⁡(*θ*_*i*_) × *e* ^−2*α*_ref_(*d*_0_/cos⁡⁡(*θ*_*i*_))^, which can be calculated by Kaiser window. The discrete probability density of Kaiser Window can be written as

(20) $$\begin{array}{c} \omega \left(m\right)=\{\begin{array}{cc} I_{0}\times \left(\pi \alpha \sqrt{1-{\left(\frac{2m}{M}-1\right)}^{2}}\right), & 0\leq m\leq M\\ 0, & \text{otherwise}, \end{array} \end{array}$$

where *U*_0_ represents the first zero-order modified Bessel function and *α* is the parameter to determine shape of the window, while *M* is an integer with length of (*N* + 1).

## 3. Experimental Results

The developed method is applied to a series of CT images obtained from PLA General Hospital to investigate the performance and accuracy of the proposed simulation algorithm. The images were acquired from a 64-slice CT scanner by Philips, and the resolution is 512 × 512 × 394. The algorithm is implemented in the C++ programming language. 

### 3.1. Evaluation of Multiscale Enhancement

**Figure 3:**
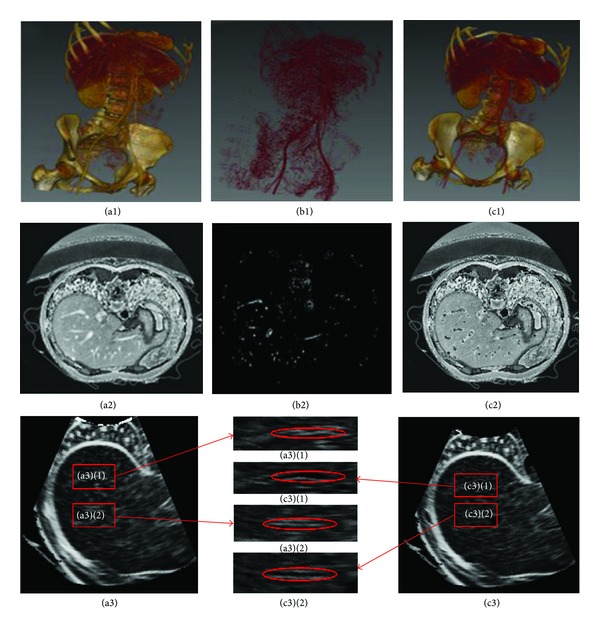
Comparison of different ultrasound simulation methods. The first row shows volume rendering of the original CT image, the extracted vascular structure, and the enhanced image, respectively. The second row shows selected liver section in transverse direction of the original CT image, the extracted vascular structure, and the enhanced image, respectively. (a3) and (c3) in the third row show simulated results of (a2) and (c2), respectively, while the middle image in the third row shows two magnified regions of interest corresponding to the same locations in (a2) and (c2).

Figure 3 shows the effectiveness of the ultrasound simulation with multiscale enhancement, which is compared with the direct simulation of the CT image. Figure 3(a1) is the volume rendering of the original image. The gray scales of vascular trees are very close to their surrounding tissues, especially for small vessel segments and bones. If ultrasound is directly simulated from this image, vessels will mix with the neighboring tissues and will be difficult to detect visually. Figure 3(b1) is the volume rendering of the vascular structure processed by the multiscale enhancement method. The vascular structures are effectively extracted from which small vessel segments can be visually inspected. Figure 3(c1) is the superimposing of the original image and the enhanced vascular structure. Evidently, the vascular trees are effectively strengthened, and they can easily be separated from the surrounding tissues. Moreover, the vascular structures can be distinguished from bones.

Figures 3(a2), 3(b2), and 3(c2) are selected section slices in the transverse direction of the original CT image, which correspond to Figures 3(a1), 3(b1), and 3(c1), respectively. Figure 3(a3) is the direct simulation result of Figure 3(a1), whereas Figure 3(c3) is the simulation result of the enhanced image in Figure 3(c1). Based on Figure 3(a2), large vascular segments and the liver have comparatively higher gray scales than their neighboring tissues, and small vessels in the liver boundaries mix with liver tissues. If the ultrasound image is directly simulated from this image, such an intensity distribution can result in a large deviation the blood flow. In Figure 3(c2), the vasculatures are filled with low intensity values, which are shown as back circle areas compared with Figure 3(c1). Figures 3(a3) and 3(c3) show the simulated results of Figures 3(a2) and 3(c2), respectively, whereas Figures 3(a3)(1), 3(c3)(1), 3(a3)(2), and 3(c3)(2) show two magnified regions of interest corresponding to the same location in Figures 3(a2) and 3(c2). Evidently, blood vessels are effectively enhanced in Figure 3(c3), which are very close to the real ultrasound images.

**Figure 4:**
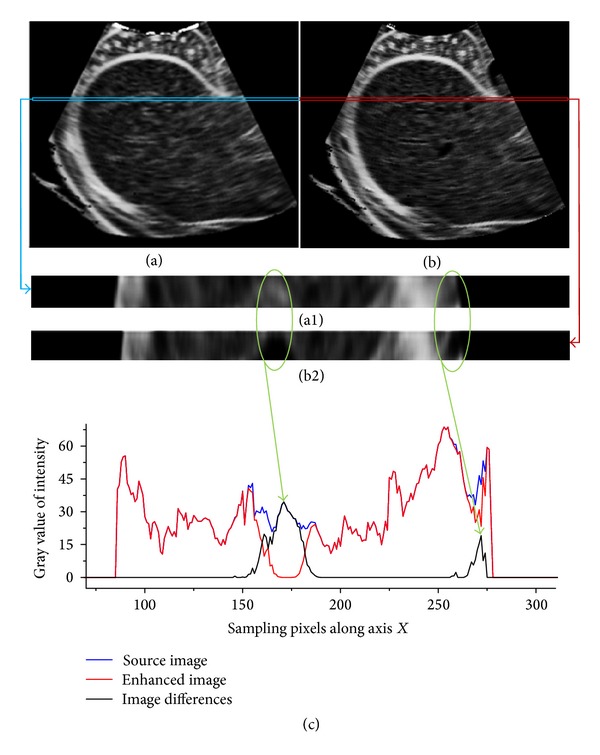
Comparison of simulated ultrasound images using direct simulation and multiscale enhancement method. (a) is the directly simulated ultrasound; (b) is the simulated result with multiscale enhancement. (a1) and (b1) show the magnified details of the rectangle strip in (a) and (b), respectively. (c) shows the intensity distribution in horizontal direction.

Figure 4 shows a comparison of simulated ultrasound images of direct simulation and multiscale enhanced simulation. Figure 4(a) is the direct simulated ultrasound, whereas Figure 4(b) is the simulated result with multiscale enhancement. Vascular structures are clearly enhanced in Figure 4(b), which are presented as a black hole in the image, and the size of the hole indicates the dimension of the vasculature. The details of the enhanced ultrasound image are also clearer than those of the direct simulated image. Figures 4(a1) and 4(b1) show the magnified details of the rectangle strip in Figures 4(a) and 4(b), respectively. Based on the ellipse areas shown in this figure, the differences between these two figures can be clearly observed. Figure 4(c) shows the intensity distribution of the selected strips of Figures 4(a) and 4(b) in the horizontal direction. The intensity difference of these two images reaches nearly 35 gray scale levels, and the location of the maximum exactly corresponds to that of the vascular structures on the *x*-axis. Clearly, direct use of the CT image as a scattering map results in a repetitive scattering pattern through which hardly any structures are recognizable. However, the tubular structure enhancement method can effectively strengthen vascular structures, and a realistic acoustic transmission pattern is simulated and visualized.

### 3.2. Multiple Transducer Elements Simulation

**Figure 5:**
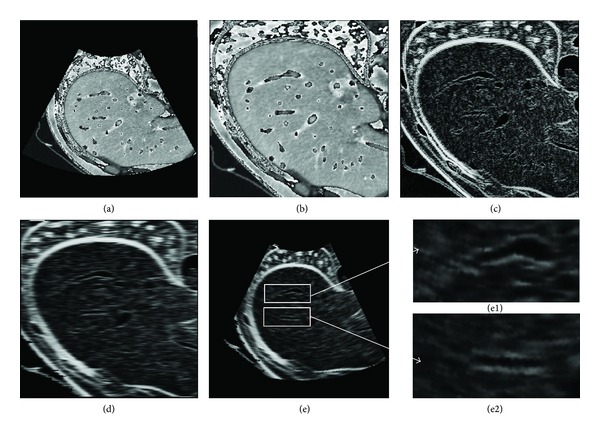
Evaluation results of multiple transducer elements simulation. (a) is the extracted sector section of the CT image. (b) is the rectangle mapping of (a). (c) is the simulated ultrasound of single transducer element. (d) is the simulated ultrasound of multiple transducer elements. (e) is the sector mapping of (d). (e1) and (e2) are the magnified regions of interest in (e).

The reflected signals of ultrasound are integrated along the active wavefront at a specified depth controlled by the Kaiser window function, which results in a more realistic reflection.  Figure 5 shows the evaluation results of the multiple transducer element simulation.  Figure 5(a) shows an extracted sector section of the CT image, Figure 5(b) gives the rectangle section image transformed by the thin-plate spline, and Figure 5(c) is the simulated ultrasound with one active element based on the acoustic transmission model, whereas Figure 5(d) is the simulated result with multiple active elements using the Kaiser window function. Figures 5(e1) and 5(e2) show two magnified regions of interest in Figure 5(e).

The thin-plate spline is very effective for the transformation of images between sector and rectangular shapes, for which smooth warping is achieved. Moreover, the highly reflective areas in the ultrasound are located around the boundary of tissues. The vasculatures can be easily identified in booth ultrasounds with single Figure 5(c) and multiple Figure 5(d) transducer elements. The difference between Figures 5(c) and 5(d) is that the edges between tissue boundaries of Figure 5(c) are significantly clearer than those of Figure 5(d). The realistic ultrasound is achieved by multiple transducer element simulation. From Figures 5(e1) and 5(e2), the vascular structures in the liver can be identified explicitly.

### 3.3. Evaluation of Ultrasound Simulation

Although a series of calculations has been applied for the simulation of ultrasound, image generation is still very efficient in terms of computation. The calculation complexity of the proposed method is decided by the sampling rate along radial and tangential directions, and it is not correlated to the FOV and the penetration depth. In order to evaluate the efficiency of the proposed method, three low cost personal PCs with different processing capacity are employed to simulate ultrasound with different sampling rates. The sampling rates are taken as 150 × 100, 200 × 150, 300 × 200, 350 × 250, 400 × 300, 450 × 350, 500 × 400, 550 × 450, and 600 × 500, while the processing platforms are as follows:

- Intel Core i5-2410 4 × 2.3 GHz, 8 G RAM, Ubuntu 12.10 (64-bit),
- Intel Core i7-860 4 × 2.8 GHz, 8 G RAM, Ubuntu 12.10 (64-bit),
- Intel Core i7-2600 4 × 3.4 GHz, 8 G RAM, Ubuntu 12.10 (64-bit).

**Figure 6:**
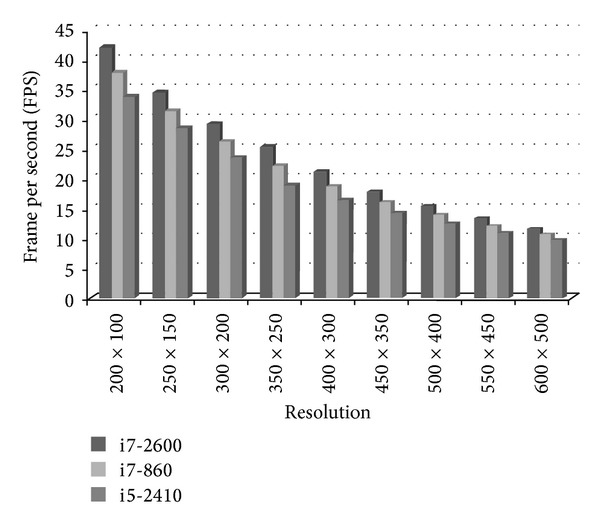
Comparison of the simulation speeds on different processing platforms.

Figure 6 compares the calculation of the frame rate of the above mentioned platforms and sampling rates. It can be seen that the calculation efficiency is reducing gradually with the increase in the sampling rate for all the platforms. When the sampling rate is 200 × 100, the calculation frame rates reaches about 42.2, 37.9, and 33.8 fps; however, when the sampling rate is about 600 × 500, the calculation frame rates are about 11.4, 10.5, and 9.6 fps. It is obvious that high performance PC can obtain fast simulation speeds.

**Figure 7:**
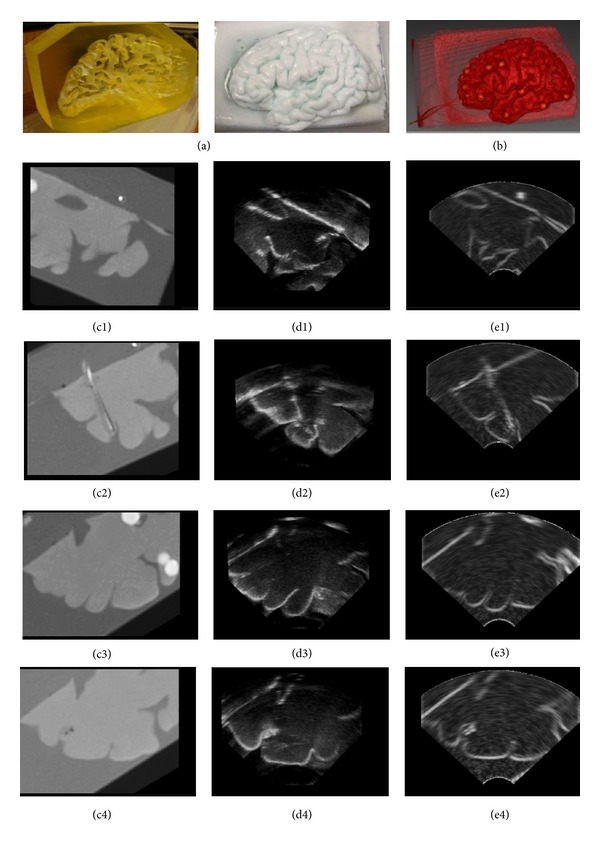
Evaluation of simulated ultrasound on phantom images. (a) Photos of the phantom. (b) Volume rendering of the CT image. ((c1)–(c4)) CT slices. ((d1)–(d4)) Ultrasound slices. ((e1)–(e4)) Simulated ultrasound sections corresponding to the CT slices.

In order to investigate the performance of the proposed ultrasound simulation algorithm, it is applied to the realistic brain phantom created from polyvinyl alcohol cryogel (PVA-C) by Chen et al. [28]. PVA-C is a material widely used in validation of image processing methods for segmentation, reconstruction, registration, and denoising for its mechanical similarities to soft tissues. The phantom was cast into a mold designed using the left hemisphere of the Colin27 brain dataset and contains deep sulci, a complete insular region, and an anatomically accurate left ventricle. The author released the CT, MRI, and ultrasound images of the phantom. All the volume data is with the size of 339 × 299 × 115, and corresponding imaging angles of ultrasound. As ultrasound and the CT images from the same imaging view can be obtained simultaneously, the fidelity of the proposed algorithm can be effectively evaluated by comparing the simulated ultrasound with the corresponding phantom.  Figure 7(a) provides photos of the elastic Colin27 based brain phantom mold and the PVA-C phantom.  Figure 7(b) gives the volume rendering of the CT image of the phantom. Figures 7(c1) to 7(c4) give the CT image slice from two different angles, while Figures 7(d1)–7(d4) provide the realistic ultrasound image of the phantom corresponding to the CT image slices. Figures 7(e1)–7(e4) give the simulation results of the CT slices by the algorithm proposed in this paper. It can be seen that our method is very effective, which obtained realistic simulation of the ultrasound image.

### 3.4. Visualization System

**Figure 8:**
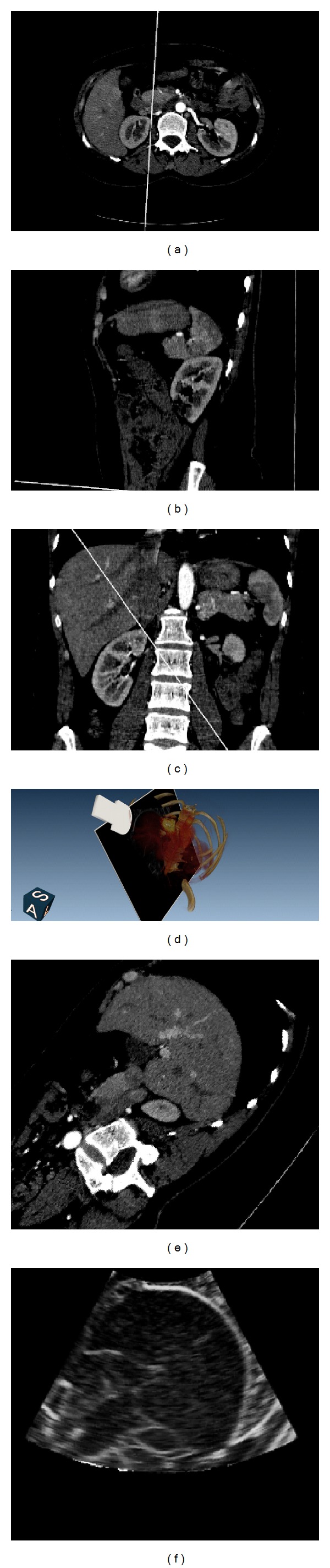
Screen-shot of the simulation system. (a), (b), and (c) are the axial plane, coronal plane and sagittal plane of CT image, respectively. (d) is the volume rendering of the CT image blended with the extracted image section on the direction and location of the virtual transducer. (e) is the extracted section of CT image. (f) is the simulated ultrasound image.

In this paper, an application system is developed for displaying the simulated ultrasound in 2D and 3D using different visualization techniques.  Figure 8 shows the screen shot of the visualization area of the developed system. The three leftmost images in this figure illustrate the axial plane in Figure 8(a), coronal plane in Figure 8(b), and sagittal plane in Figure 8(c) on the normal direction of the ultrasound transducer. The top right figure shows the volume rendering of the original CT image in Figure 8(d), whereas Figure 8(e) shows the extracted section plane of the CT image, and Figure 8(f) is the simulated ultrasound. Based on this system, the ultrasound image is generated according to the location and direction of the transducer. The ultrasound and volume rendering of the CT image can be displayed with the three orthographic views of the CT image. Based on this system, the ultrasound image is fast generated, and the parameters, including ultrasound simulation and visualization, can be adjusted from user interface interaction.

The developed simulation system comprises four main visualization function modules, as follows. (1) The position and orientation of the virtual probe can be interactively set by dragging the mouse in the 3D or the three orthogonal views, whereas the FOV, minimum, and maximum PD can be adjusted in the control panel. (2) The transparency and color mapping of volume rendering can be adjusted by controlling the multipoint thresholds on the histogram distribution. (3) The window level and window width for the CT slice in different views can be adjusted simultaneously using the slider bar. (4) Each view in the display window can be maximized to full screen model and reset to its default.

**Figure 9:**
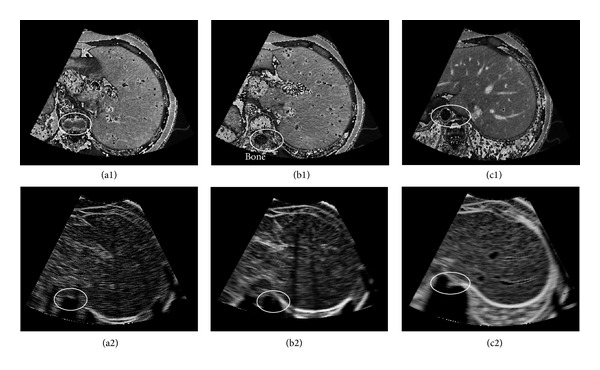
Simulation results of the proposed algorithm. The first row is the sector area of the CT slice. The second row is the simulated ultrasound.

Figure 9 gives the final simulation results of three sections of abdominal CT images. The first row shows the extracted sector CT image, while the second row gives the corresponding simulated ultrasound. It can be seen that the internal structure of the liver can be visualized clearly. In the CT slices, the spines can be visually detected in the left bottom parts, as marked in the circles. In the simulated images, it can be seen that lower parts away from the spines are displayed as black empty areas. Obviously, the acoustic wave is absorbed by the bones and cannot be transmitted to the lower parts of the simulated images. Our algorithm effectively simulated the ultrasonic propagation phenomenon.

## 4. Conclusion and Discussion

The ultrasound simulation technique not only provides a cheap and efficient way of training doctors in the study of the anatomic structure of human body but can also be used to validate the registration efficiency of the ultrasound navigation system. In this paper, a novel framework is proposed for fast ultrasound simulation and visualization. A multiscale method is utilized to enhance the tubular structure of the CT image and to obtain a realistic simulation of the vascular structure. Seamless transformations between sector and rectangle shapes are then achieved using the thin-plate spline interpolation method. The parameters of acoustic response are based on the intensity difference ratio of adjacent regions for acoustic wave propagation in a piecewise homogenous medium and are fast calculated. Moreover, the detected edge information on different tissues is combined with random noises to simulate the acoustic response rate of the interesting region. Speckle noise and blurring are also added to the simulated ultrasound, resulting in an image that can be fast updated according to the user-defined parameters. Finally, the Kaiser window function is employed to simulate integration effects of multiple transducer elements. Based on the experimental results, realistic simulation results are obtained. Aside from soft tissues and bones, vasculatures can be clearly observed in the simulated ultrasound. Based on the efficiency evaluation experiments, the proposed simulation method is also very fast. The average frame rate of the proposed ultrasound simulator is approximately 20 fps (SM = 300, FOV = 75°), which is better than the 16 fps rate commonly used in clinical radiology. However, the quantitative evaluation of the ultrasound simulation techniques is very difficult so far because of three main reasons: first, it is difficult to obtain the accurate imaging angle of the handheld ultrasound probe. Second, it is very difficult to control the pressure degree on soft tissues during the imaging procedures, for which a different pressure will lead to a different imaging depth. Third, the imaging quality of the ultrasound is strictly correlated with the adjustable parameters of the transducer elements. Hence, it is very difficult to obtain the ultrasound with predefined imaging parameters, which hence can be evaluated from the anatomic structures in CT image. Up to now, the commonly used evaluation method for ultrasound simulation is the visual comparison by physicians in clinical practice. In this paper, the effectiveness of the developed method is quantified on realistic brain phantoms. And the experimental results are assessed by experts from the ultrasonic department at the General Hospital of People's Liberation Army, China.

The interesting application of the proposed method is its use in training for different ultrasound examinations or ultrasound-guided procedures. During a training session, the simulated ultrasound can be displayed with the model constructed from the CT image to provide an anatomical context to the trainee. Vascular enhancement and scattering image simulation are time consuming and require a cluster of CPUs to be practical. Hence, GPU implementation of the algorithm will considerably accelerate the simulation speed, which will meet the higher requirements of fine-resolution simulation. In this paper, all acquisition parameters can be interactively adjusted during simulation processing, including ultrasound frequency, ultrasound intensity, FOV, PD, as well as speckle noise size. Hence, the proposed simulation method is highly convenient for the simulation of different imaging conditions.
